# Low Dose Influenza Virus Challenge in the Ferret Leads to Increased Virus Shedding and Greater Sensitivity to Oseltamivir

**DOI:** 10.1371/journal.pone.0094090

**Published:** 2014-04-07

**Authors:** Anthony C. Marriott, Brian K. Dove, Catherine J. Whittaker, Christine Bruce, Kathryn A. Ryan, Thomas J. Bean, Emma Rayner, Geoff Pearson, Irene Taylor, Stuart Dowall, Jenna Plank, Edmund Newman, Wendy S. Barclay, Nigel J. Dimmock, Andrew J. Easton, Bassam Hallis, Nigel J. Silman, Miles W. Carroll

**Affiliations:** 1 Public Health England, Salisbury, United Kingdom; 2 Division of Infectious Disease, Imperial College London, London, United Kingdom; 3 School of Life Sciences, University of Warwick, Coventry, United Kingdom; University of Liverpool, United Kingdom

## Abstract

Ferrets are widely used to study human influenza virus infection. Their airway physiology and cell receptor distribution makes them ideal for the analysis of pathogenesis and virus transmission, and for testing the efficacy of anti-influenza interventions and vaccines. The 2009 pandemic influenza virus (H1N1pdm09) induces mild to moderate respiratory disease in infected ferrets, following inoculation with 10^6^ plaque-forming units (pfu) of virus. We have demonstrated that reducing the challenge dose to 10^2^ pfu delays the onset of clinical signs by 1 day, and results in a modest reduction in clinical signs, and a less rapid nasal cavity innate immune response. There was also a delay in virus production in the upper respiratory tract, this was up to 9-fold greater and virus shedding was prolonged. Progression of infection to the lower respiratory tract was not noticeably delayed by the reduction in virus challenge. A dose of 10^4^ pfu gave an infection that was intermediate between those of the 10^6^ pfu and 10^2^ pfu doses. To address the hypothesis that using a more authentic low challenge dose would facilitate a more sensitive model for antiviral efficacy, we used the well-known neuraminidase inhibitor, oseltamivir. Oseltamivir-treated and untreated ferrets were challenged with high (10^6^ pfu) and low (10^2^ pfu) doses of influenza H1N1pdm09 virus. The low dose treated ferrets showed significant delays in innate immune response and virus shedding, delayed onset of pathological changes in the nasal cavity, and reduced pathological changes and viral RNA load in the lung, relative to untreated ferrets. Importantly, these observations were not seen in treated animals when the high dose challenge was used. In summary, low dose challenge gives a disease that more closely parallels the disease parameters of human influenza infection, and provides an improved pre-clinical model for the assessment of influenza therapeutics, and potentially, influenza vaccines.

## Introduction

In April 2009, a novel H1N1 influenza A virus of swine origin emerged from North America and spread around the world, resulting in the first influenza pandemic of the 21^st^ century [1]. The pandemic H1N1 virus (H1N1pdm09) contained a unique constellation of genes derived from North American triple-reassortant swine influenza and Eurasian swine influenza, and was antigenically unrelated to the seasonal H1N1 virus circulating in the human population prior to that time [2]. Infection resulted predominantly in a mild disease, but developed into severe illness associated with mortality in a minority of cases [3], [4]; the global mortality has been estimated as 300,000 [5]. H1N1pdm09 virus is now established worldwide as a seasonal infection, and has fully replaced the previous seasonal H1N1 virus.

The ferret model for influenza infection is often referred to as the “gold standard” model since these animals display several key attributes considered to be predictive of disease severity observed in human influenza infection [6], [7]. Ferrets are susceptible to human influenza virus isolates without requiring prior adaptation of the viruses, develop clinical signs similar to those in human infections (such as fever, sneezing and lethargy), possess a similar respiratory tract physiology, and have α-2,6 and α-2,3-linked sialic acid virus receptors with a distribution similar to that in the human respiratory tract [8]. Infection of ferrets via the intra-nasal route with H1N1pdm09 virus has typically induced a mild to moderate, nonlethal infection resulting in weight loss, transient pyrexia, and mild upper respiratory tract signs. These studies have typically used a dose of 10^6^ TCID_50_ per ferret in order to ensure all ferrets become infected [9]–[12]. However this dose is a gross exaggeration of the dose which is required to infect humans (0.6–3 TCID_50_ by aerosol challenge) [13], [14], and ferrets are susceptible to much lower doses of influenza viruses than 10^6^ TCID_50_. For example, 50% ferret infectious doses (FID_50_) have been estimated to be as low as 10–30 TCID_50_ for pre-pandemic H1N1 viruses, 3 plaque-forming units (pfu) for a ferret-adapted H1N1pdm virus, 1–2 pfu for an H3N2 virus and 4 pfu for an H5N1 virus [15]–[17]. Use of high dose inocula may enhance pathogenicity and accelerate infection kinetics, which may obscure the effects of antiviral interventions. It has been shown, for example, that a high dose of influenza A virus (10^6.8^ EID_50_) overcame the protective effect of a defective interfering virus preparation, which was fully protective in mice when a lower challenge dose (10^3.5^ EID_50_ virus) was used [18]. This consideration is particularly relevant for the ferret model of influenza, as these animals have been used extensively in demonstrating the efficacy of anti-influenza drugs such as the neuraminidase inhibitors oseltamivir, zanamivir and peramivir [12], [19]–[23].

The A/California/04/2009 isolate of H1N1pdm09 virus (Cal/04) was isolated early in the pandemic from a non-fatal infection in a 10 year old boy [24]. When compared to other H1N1pdm09 isolates in the ferret model, Cal/04 induced only mild disease, and is thus a suitable model virus for typical human H1N1pdm09 infections [9], [25]. A challenge dose of only 100 pfu Cal/04 has been used previously in our laboratory to infect ferrets intra-nasally [26], [27]. The purpose of this study was to further characterize the influenza infection model using a low dose (100 pfu) of the Cal/04 strain, to compare the kinetics and severity of infection with this dose to higher doses (10^4^ or 10^6^ pfu) delivered by the same route, and to determine if the size of infectious dose had an effect on the observed antiviral activity of standard oseltamivir treatments.

## Methods

### Animals

Ferrets (*Mustela putorius furo*) were obtained from Highgate Farm, UK, and confirmed as seronegative for influenza H1N1pdm09 antibodies by haemagglutination-inhibition assay before experiments commenced. Mean weight at challenge was approximately 950 g (range 707–1367 g), and either females (3 experiments; age range 6–12 months) or males (1 experiment; age range 3–4 months) were used. An identifier chip (iDENTICHIP, Bio-Thermo) was inserted subcutaneously into the dorsal cervical region of each animal. Animals were monitored for signs of disease twice daily (approximately 8 hr apart), and weight and temperature were recorded once or twice daily, respectively. Animals were sedated by intramuscular injection of ketamine/xylazine (17.9 mg/kg and 3.6 mg/kg bodyweight), prior to intranasal instillation of challenge virus (routinely 0.2 ml total, 0.1 ml per nostril) diluted in phosphate buffered saline (PBS). Nasal washes were obtained using 2 ml PBS [28]. At various times post-infection, ferrets were anaesthetised, exsanguinated by cardiac puncture, and tissue samples were collected. The experimental animal work described here was scrutinized and approved by the Animal Welfare and Ethical Review Body of Public Health England (Porton), as required by the UK Home Office Animals (Scientific Procedures) Act, 1986. The premises in which the work was conducted are approved under Home Office Certificate of Designation PCD70/1707.

### Clinical scoring

The most frequently observed clinical signs of disease were sneezing, nasal discharge and inactivity (lethargy), each of which was monitored twice daily. For each ferret in each observation period, a score of 0 or 1 was assigned for the absence or presence, respectively, of sneezing or nasal discharge. Activity was scored as 0 =  normal activity, 1 =  reduced activity, and 2 =  inactive. All scores were summed for each ferret over the period of observation (starting from the day of inoculation), divided by the number of days, and averaged for the treatment group to give a mean score per ferret per day. Viable cells in nasal washes were counted by addition of 1/10 volume 0.4% Trypan Blue (Sigma), using a haemocytometer.

### Virus

Influenza A/California/04/09 (H1N1) was obtained from the Centers for Disease Control and Prevention (CDC, Atlanta, USA), and propagated in Madin-Darby Canine Kidney (MDCK) cells, obtained from European Collection of Cell Cultures (ECACC, Porton Down, UK). The identity of the virus was confirmed by sequencing the HA and NA genes. Virus titres were determined by plaque assay on MDCK cells under an agar overlay, followed by staining with crystal violet.

### Oseltamivir treatment

In two experiments, ferrets were treated with oseltamivir (Tamiflu, Roche). The drug was dissolved in sterile water to 12 mg/ml, and delivered to ferrets by oral gavage twice daily. Doses of 5 mg/kg/day and 10 mg/kg/day are equivalent to human doses of 75 and 150 mg/day, respectively [29], and represent prophylactic and therapeutic doses. Prophylactic dosing commenced 2 hr prior to infection; therapeutic dosing commenced 6 hr post-infection. In both experiments, dosing continued for 5 days.

### Quantitative real-time reverse transcriptase PCR (qRT-PCR)

Total RNA was extracted from ferret tissues, which had been collected into RNALater (Qiagen) and stored at −20°C (lung and trachea), or frozen at −80°C (nasal turbinate), using the Qiagen RNeasy Mini kit (lung) or RNeasy Fibrous kit (trachea, nasal turbinates). Lung samples were taken from the upper left lobe in each case. RNA was quantified spectrophotometrically using a Nanodrop ND-1000. RNA quality was assessed using an Agilent 2100 Bioanalyzer. Absolute quantification of influenza virus segment 7 was determined using a quantified, negative-sense synthetic T7 RNA polymerase transcript, from a full-length plasmid clone of Cal/04 segment 7, to construct a standard curve. Reactions used primers (M+24, M-124Mod) and probe (M+64 with 5′-FAM and 3′-BHQ1) as described [30], [31] with the Superscript III Platinum One-Step qRT-PCR kit (Invitrogen), and were analysed using the ABI Prism 7900HT and SDS 2.4 software (Applied Biosystems).

### Pathological studies

Samples of nasal cavity, trachea and lung were fixed in 10% neutral buffered formalin, and processed routinely to paraffin wax. Sections of 4–6 μm were stained with haematoxylin and eosin (HE) for examination by light microscopy. For the therapeutic oseltamivir study, the severity of changes in each section was scored subjectively, as minimal, mild, moderate, or marked. The sections were examined “blind” to eliminate observer bias.

### Statistical methods

Advice was obtained from a qualified statistician. Differences between groups were assessed for statistical significance using the Mann-Whitney U-test at a level of *p*<0.05. Means of logarithmic data were calculated and plotted as geometric mean. Area under the curve was calculated by numerical integration using the trapezoidal rule.

## Results

### Low dose challenge leads to delayed clinical disease kinetics and increases virus shedding

**Figure 1:**
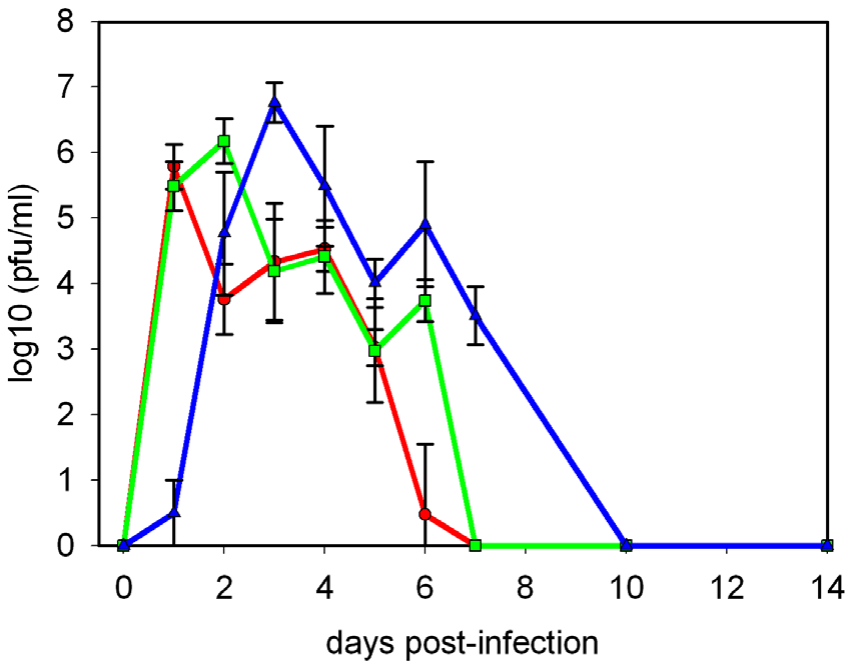
Effect of decreasing infectious dose on virus shedding. Ferrets were infected intra-nasally, and nasal washes were collected at the intervals shown for virus plaque assay. Markers show geometric mean nasal wash titre from groups of 5 or 8 ferrets; error bars show standard deviation. For days 10 and 14 post-infection, 2 ferrets per group were used. • (red) high dose (10^6^ pfu), ▪ (green) medium dose (104 pfu), ▴ (blue) low dose (102 pfu) inoculum. The lower limit of detection was 10 pfu/ml.

**Figure 2:**
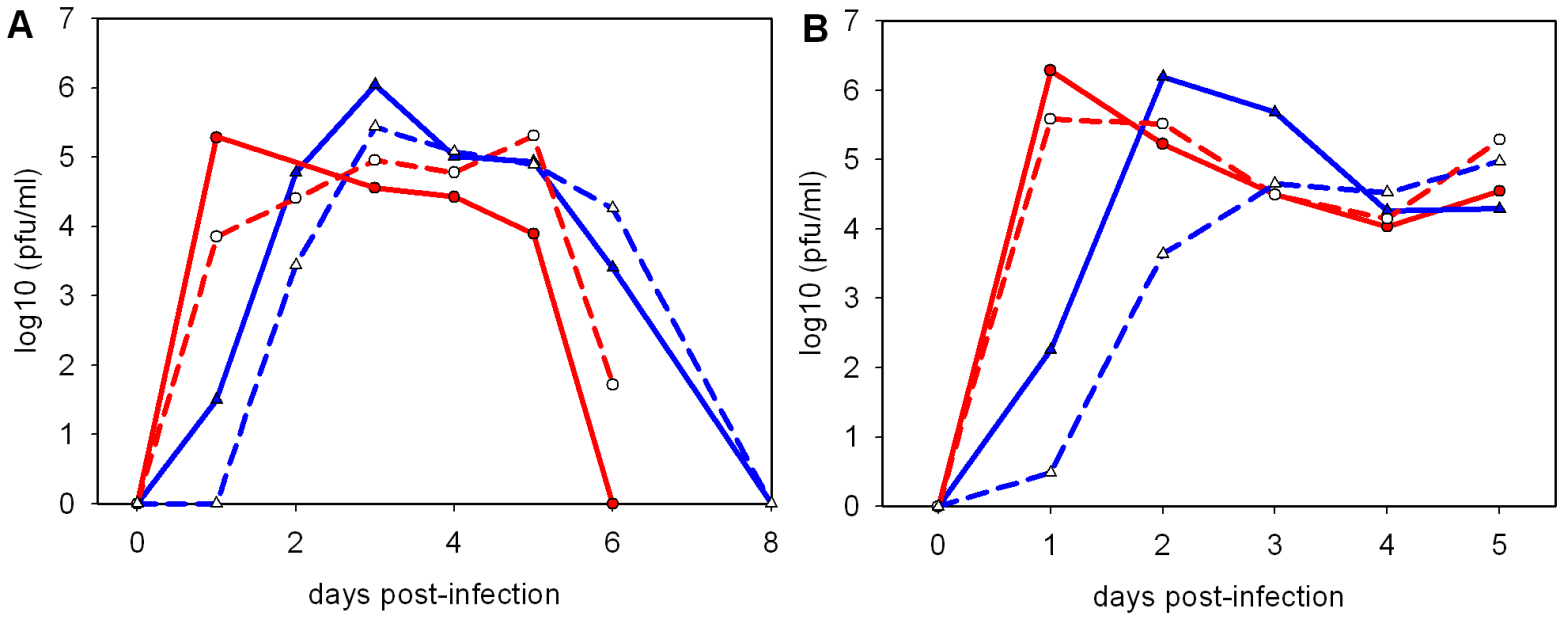
Nasal wash virus titres in the presence or absence of treatment with oseltamivir. A, prophylactic oseltamivir regimen from 2; B, therapeutic oseltamivir regimen from 6 hr post-infection. • (red) high dose (10^6^ pfu); ▴ (blue) low dose (10^2^ pfu); open symbols represent oseltamivir-treated animals. Means from 5 ferrets (A) or 3–9 ferrets per group (B).

Ferrets were infected with Cal/04 by the intra-nasal route with the widely used high dose (10^6^ pfu per ferret), a medium dose (10^4^ pfu), or a low dose (10^2^ pfu), and disease progression was monitored for up to 14 days post-infection (dpi). The results of the clinical findings are shown in Table 1. All challenged animals demonstrated clinical signs of disease, which were not observed in mock-infected animals, including transiently elevated temperature, transient weight loss, sneezing, nasal discharge, and inactivity, although not all signs were observed in every animal. The most notable effect of reducing the virus inoculum from 10^6^ pfu to 10^2^ pfu was to delay the progression of disease by approximately 1 day, with a dose of 10^4^ pfu giving an intermediate delay. This delay encompassed peak temperature, weight loss, and onset of clinical signs (Table 1). Shedding of virus into nasal washes was also delayed by approximately one day for each 100-fold reduction in virus dose (Fig. 1). Virus clearance (as determined by lack of detectable infectious virus in nasal wash liquid) was also delayed in ferrets receiving lower doses. With high and medium doses, virus was undetectable in nasal washings by 7 dpi, compared to 10 dpi for low dose infection (Fig. 1). It was also notable that the peak virus titre was not reduced in animals receiving lower doses, and in 2 out of 3 studies the peak titre of virus shed by the low-dose animals was significantly higher (5 to 9-fold) than the peak of virus shed by high-dose animals (Figs. 1 and 2). Using area-under-the-curve as a marker for total virus shedding, the low dose infected animals shed 53% more virus than the high dose infected animals, with those infected with 10^4^ pfu being intermediate. The 1 day delay seen in disease progression when infectious dose was reduced to 10^2^ pfu was reproducible with both male and female ferrets of different ages (between 3 and 12 months).

**Table 1:** Effect of virus dose on parameters of infection with influenza A/California/04/09.

Infecting dose (pfu)	10^6^	10^4^	10^2^	mock
**Day of peak temperature**	2	2	3	none
**Day of greatest weight loss**	2	3	3	none
**Day of maximum nasal wash cell count**	2	ND	3	none
**Mean clinical score** 1	1.48±0.35	1.08±0.46	0.79±0.07	0.00
**Day of onset of clinical signs** 2	2	2	3	none
**Day of peak virus shedding**	1	2	2–3	none

1 Mean clinical score is calculated as described in Materials and Methods, and expressed as mean score per ferret per day ± standard error of the mean.

2 Day of onset refers to median onset of respiratory signs and inactivity from four studies.

ND, not done.

### Low dose challenge leads to delayed, but not reduced, pathological changes in the respiratory tract

Overall the pathological changes observed were similar to previous studies in ferrets using 10^6^ pfu [10], [11], [32] or 10^2^ pfu [33] of H1N1pdm09 virus.

At 2 days post-challenge, there was multifocal epithelial necrosis and sloughing, with a mixed, mainly polymorphonuclear inflammatory cell infiltrate, in the nasal cavity of animals that received a dose of 10^6^ or 10^4^ pfu Cal/04. By contrast, the nasal cavity of the low dose group animals (10^2^ pfu), was either normal or contained only a mild, mononuclear cell infiltrate. In the lung, in all dose groups, bronchiolar inflammatory cell exudation and focal, parenchymal, mononuclear cell infiltrates with a dose dependent increase in severity, were seen in six animals (2 out of 5 animals per group). In addition, variable necrotising bronchiolitis and bronchial gland necrosis were noted between the groups.

By 5–7 dpi, in the nasal cavity, there was extensive epithelial loss with attenuation of remaining epithelial cells, and marked, suppurative exudation, with similar severity in all animals at all doses. In addition, in 9 out of 9 animals examined at 7 dpi, regenerative changes in the epithelium were observed comprising hypertrophy, and hyperplasia with basophilic cytoplasm. In the trachea, mild changes including mucosal inflammation and glandular necrosis were seen in some animals in the high and low dose groups, however, changes were not seen in 4 out of 4 animals examined in the mid dose group. In the lung, necrotising bronchiolitis was seen in 3 out of 5 animals in the low dose group, and all animals in the mid and high dose groups. Bronchial gland necrosis was seen in all animals in all dose groups. In addition, perivascular oedema was observed in 3 out of 5 animals in the high dose group.

By 14–15 dpi, lesions in the nasal cavity were resolving in the mid and high dose groups but some acute inflammation and epithelial hypertrophy and hyperplasia were still present in the low dose group. In the lower respiratory tract, minimal, resolving changes and mild, residual inflammatory changes were noted in the majority of animals at all doses.

### Low dose challenge shows increased sensitivity to treatment with oseltamivir

**Figure 3:**
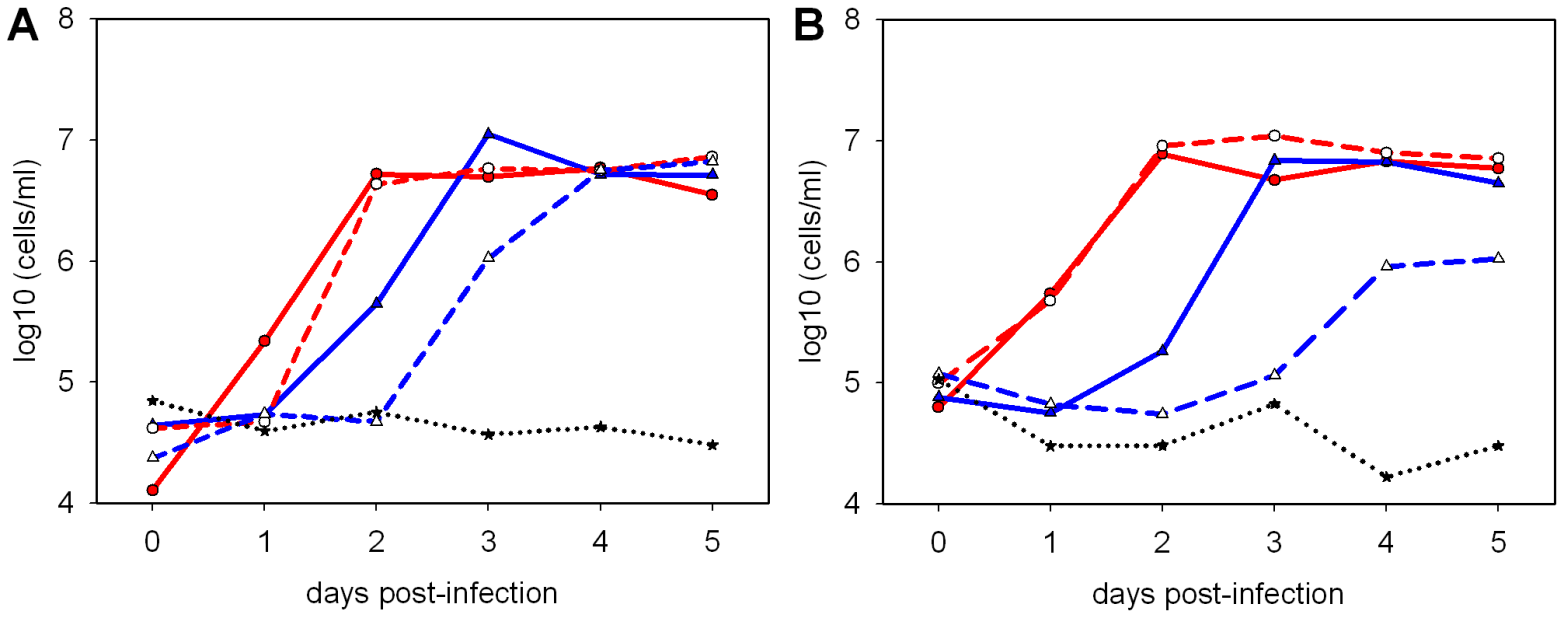
Nasal wash cell counts in the presence or absence of oseltamivir treatment. A, prophylactic oseltamivir regimen from 2; B, therapeutic oseltamivir regimen from 6 hr post-infection. • (red) high dose (10^6^ pfu); ▴ (blue) low dose (10^2^ pfu); ★ mock infected animals; open symbols represent oseltamivir-treated animals. Means from 5 ferrets (A) or 3–9 ferrets per group (B).

Since a dose of 10^2^ pfu was sufficient to reproducibly induce disease and virus shedding from infected ferrets, the effect of oseltamivir treatment on animals infected with high (10^6^ pfu) or low (10^2^ pfu) doses of Cal/04 was compared. As a marker of innate immune response to infection immune cells in nasal wash fluid were enumerated (Fig. 3) [34]. Nasal wash cells rose from a baseline of approximately 10^5^ cells/ml and reached a similar plateau value of approximately 10^7^ cells/ml in both high and low dose infection (Fig. 3, A and B). However the low dose group showed a delay of 1 day compared to the high dose group in reaching maximum concentration. Cell counts in the high dose group were significantly above baseline by 1 dpi (p<0.05), whereas in the low dose group cell counts did not rise significantly until 2 dpi. Treatment with oseltamivir had little effect on nasal wash cells in the high dose group and did not depend on whether treatment started before (Fig. 3A) or after (Fig. 3B) infection. However for the low dose group, oseltamivir treatment led to a delay in the increase of nasal wash cells, with significant reductions in cell counts from treated ferrets on days 2 and 3 post-infection (Figs. 3A and 3B) (Mann-Whitney U-test, p<0.05). The effects of oseltamivir treatment on the clinical progress of infection were not statistically significant in the high dose group, but for some parameters were statistically significant in the low dose group, and these are summarized in Table 2. In the therapeutic study, clinical signs were not observed in the oseltamivir-treated low dose challenge group. A significant reduction in peak temperature of 0.5°C due to therapeutic (but not prophylactic) oseltamivir treatment was observed in the low dose group, but not in the high dose group (Table 2).

**Table 2:** Effect of oseltamivir on clinical parameters following infection with high and low doses of influenza A/California/04/09.

	Infecting dose (pfu)	
Effect of oseltamivir on:	10^6^	10^2^
**Nasal wash cell count**	Reduction on day 1^1^ or no effect^2^	Delayed rise^1^^,^ 2; reduction on days 2^1^ and 3^1^^,^ 2
**Weight loss**	No significant effect	No significant effect
**Peak temperature**	No significant effect	Significant reduction day 3^2^ or no effect^1^
**Clinical score**	No significant effect	Score reduced to zero^2^ or no effect^1^
**Day of onset of clinical signs**	No change	No change^1^ or no signs^2^

1 Prophylactic dose of oseltamivir.

2 Therapeutic dose of oseltamivir. No effect implies not statistically significant (Mann-Whitney test, p>0.05).

Prophylactic and therapeutic oseltamivir treatments also both resulted in a significant reduction in virus shedding only with the low dose challenge (Fig. 2B). There were significant reductions in mean titre at 2 dpi (prophylactic oseltamivir, Fig. 2A, Mann-Whitney U-test, p<0.05) or 1, 2 and 3 dpi (therapeutic oseltamivir, Fig. 2B). The reduction in mean titre observed in the high dose group at 1 dpi in Fig. 2A was not significant.

### Oseltamivir leads to reduced lung virus load only with low dose challenge, and reduced trachea virus load with either challenge dose

**Figure 4:**
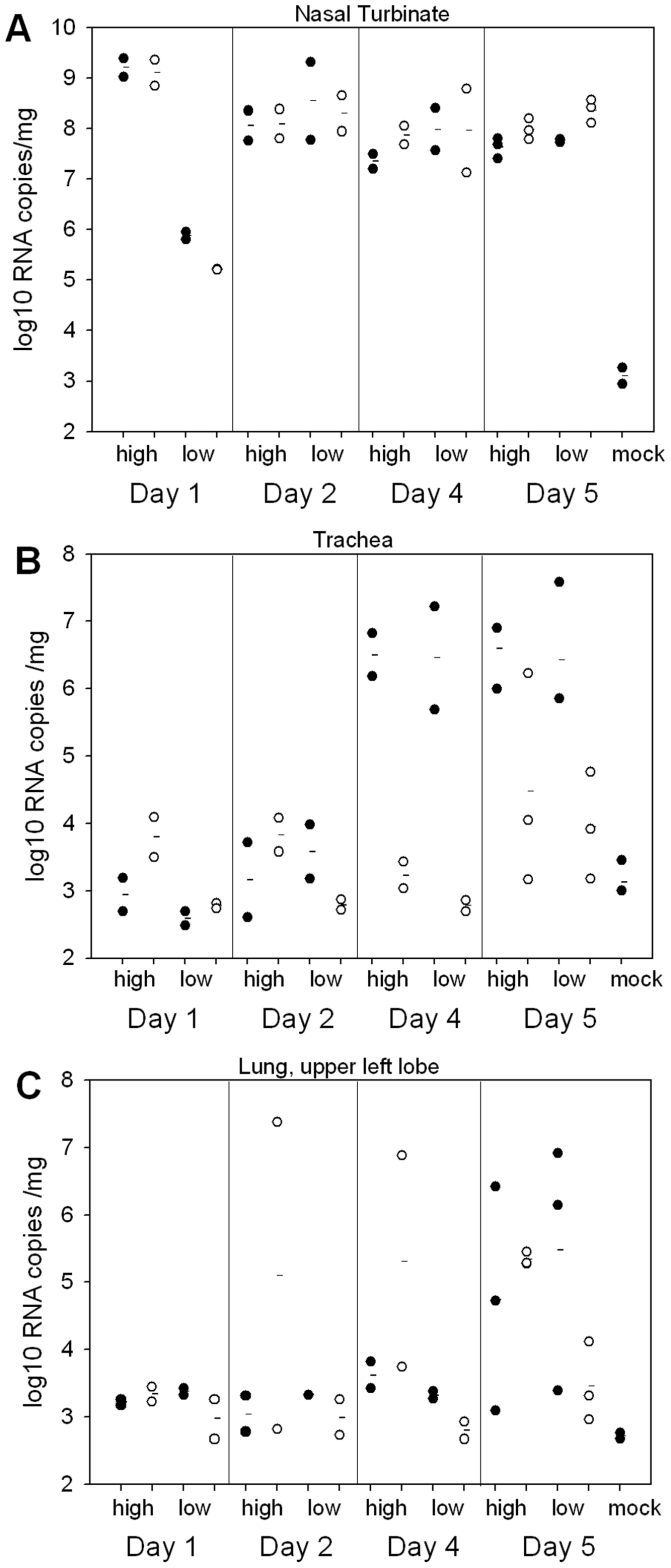
Viral RNA loads in ferret respiratory tract tissues. Ferrets were infected intra-nasally with 10^6^ or 10^2^ pfu Cal/04 and, where indicated, treated with oseltamivir from 6 hr post-infection. Circles show RNA loads for individual animals. Horizontal lines show group means. Filled circles, no treatment; open circles, oseltamivir treated. A, nasal turbinate; B, trachea; C, lung. High, 10^6^ pfu inoculum; Low, 10^2^ pfu inoculum. Samples were taken from 2 ferrets on days 1 to 4, and 3 ferrets on day 5. The sensitivity of the assay was approximately 10^3^ copies/mg.

In order to further investigate the effects of oseltamivir treatment on ferrets infected with a high or low dose of virus, samples of upper respiratory tract (nasal turbinate) and lower respiratory tract (trachea, lung) were collected for viral load analysis at 1, 2, 4 and 5 dpi. Initial experiments using Cal/04 infection indicated that although significant amounts of infectious virus could be recovered from nasal turbinates (≥10^7^ pfu/g at 2 dpi), recovery from lower respiratory tract tissues was low and variable (data not shown). Hence we opted to determine virus RNA load in tissues by real-time qRT-PCR. Nasal turbinates gave a peak titre on day 1 post-infection for the high dose group (of ∼10^9^ copies/mg of tissue) and at 2 dpi for the low dose group (∼10^8^ copies/mg of tissue) (Fig. 4A). These timings correspond to the peaks observed in nasal wash virus shedding (Figs. 1 and 2). There was little effect of oseltamivir on nasal turbinate RNA load, although the 1 dpi low dose challenge ferrets showed a mean 5-fold reduction in oseltamivir-treated animals compared to untreated animals. Nasal turbinate RNA levels remained high in all groups at least until day 5 (Fig. 4A). In the trachea, peak RNA loads of >10^6^ copies/mg were observed by 4 dpi. Both high and low dose groups showed >1000-fold reduction in virus RNA copy number due to oseltamivir at 4 dpi, and 100-300-fold reduction at 5 dpi (Fig. 4B). Noticeably, in the lung tissues of the low dose group, there was a reduction observed at 5 dpi of 100-fold in mean RNA load due to oseltamivir treatment, which was not observed in the high dose group (Fig. 4C). The baseline lung RNA loads observed on day 1 (8/8 ferrets) and day 2 pi (7/8 ferrets) suggest that direct delivery of inoculum to the lung was not a problem in this particular study. One factor in avoiding such a problem is the use of a small inoculum volume (0.2 ml per animal) in this study, as opposed to 0.5–1 ml which is often used in ferret challenge studies. Not all untreated ferrets showed a high lung RNA load by day 5 (2/3 in each of the low dose and high dose groups, for the upper left lobes which were tested), indicating some variability between animals. This variability was also reflected in two ferrets in the high dose, oseltamivir-treated group showing RNA loads of ≥10^7^ copies/mg at 2 and 3 dpi, respectively, accounting for the greater mean RNA loads in lung on these days (Fig. 4C).

### Oseltamivir treatment following low dose challenge, leads to delayed viral-induced pathological changes in the nasal cavity, and reduced changes in the lung

**Figure 5:**
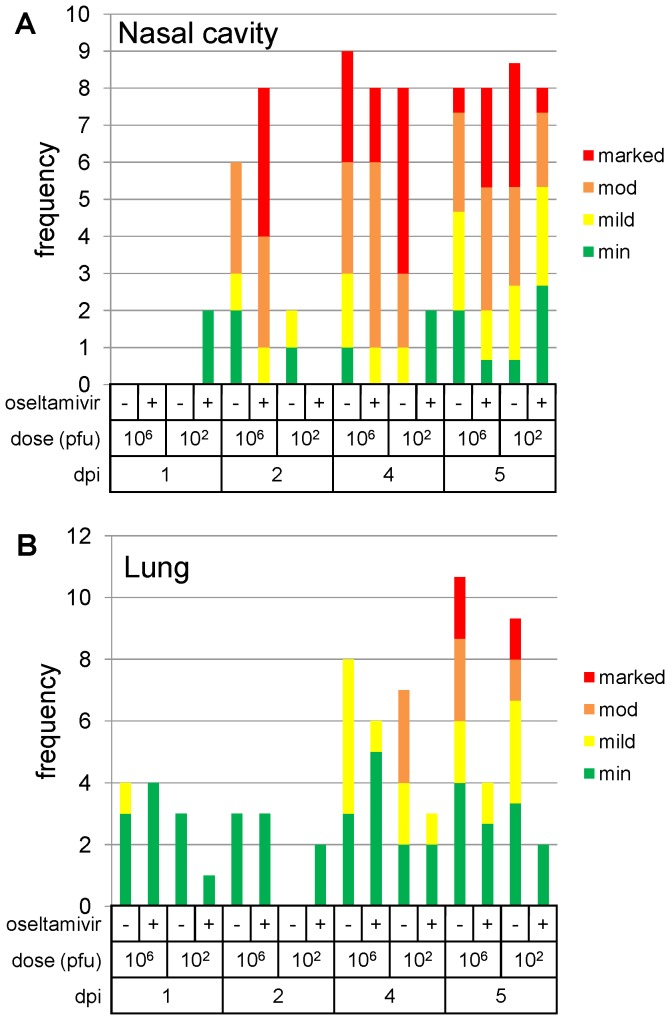
Summary of severity of pathological changes in untreated or oseltamivir treated ferret tissues. A. Nasal cavity, B. lung. In each case, changes were scored as minimal (min), mild, moderate (mod), or marked, and were summed for each group of ferrets on each day post-infection. Group size was 2 ferrets, except day 5 which was groups of 3 ferrets. The day 5 summed frequencies have been normalised to facilitate comparison to the other days.

Pathological changes in the nasal cavity and lung are summarized graphically in Figure 5. In the nasal cavity, significant changes were not observed until 2 dpi. Epithelial loss, necrosis and attenuation; inflammatory cell infiltration and oedema of the propria mucosa; and a suppurative exudate, were present. These changes were more severe (Fig. 5A) in the high dose, oseltamivir-treated group, with decreasing severity in the high and low dose, untreated groups, respectively. Changes were not observed in the low dose, oseltamivir-treated group.

**Figure 6:**
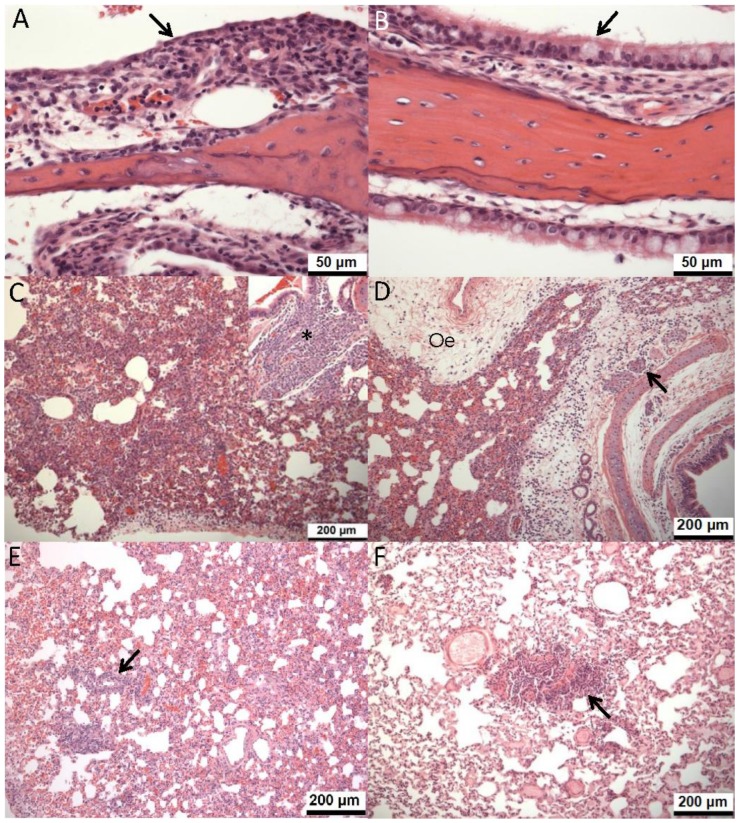
Microscopic changes in ferret nasal cavity and lung, with and without oseltamivir treatment. A. Nasal cavity, low dose, untreated group, 4 dpi. Propria mucosa is infiltrated by marked numbers of mixed inflammatory cells. Overlying respiratory epithelium comprises attenuated, pre-ciliated, regenerating cells (arrow). B. Nasal cavity, low dose with oseltamivir-treated group, 4 dpi. A mild, mononuclear cell infiltrate within the nasal propria mucosa underlying a normal, pseudostratified, columnar, ciliated epithelium (arrow). C. Lung, low dose, untreated group, 5 dpi. Multifocally extensive, mononuclear cell infiltration of parenchyma. Inset: focal, bronchial gland necrosis (asterisk). D. Lung, high dose, untreated group, 5 dpi. Marked infiltration of parenchyma with inflammatory cells by peribronchial and perivascular oedema (Oe) and bronchial gland necrosis (arrow). E. Lung, low dose, oseltamivir-treated group, 5 dpi. Minimal, parenchymal, mononuclear cell infiltrate (arrow). F. Lung, high dose, oseltamivir-treated group, 5 dpi. Mild, peribronchiolar, mononuclear cell infiltration (arrow). Haematoxylin and eosin.

On 4 dpi, similar changes to those described above, and of similar severity to changes in the high dose, oseltamivir-treated group 2 dpi, were seen in the high dose, oseltamivir-treated and untreated groups, and the low dose, untreated group (Figs. 6A). In 2 animals in the low dose, oseltamivir-treated group, only minimal inflammatory cell infiltration of the propria mucosa was observed (Fig. 6B). Regenerative changes in the surface epithelium, were also observed in the high dose, oseltamivir-treated and untreated groups, and 1 of 2 animals in low dose, untreated group. They were not seen in the low-dose, oseltamivir-treated group.

By 5 dpi, acute and regenerative changes, described above, were observed in all groups and of similar severity.

In the trachea, significant changes were not observed until 4 dpi, and comprised proprial mucosal gland necrosis in only one animal in the high dose, and one animal in the low dose, untreated groups. On day 5 pi, minimal glandular necrosis and mild, inflammatory cell infiltration were observed in 1 animal in the high dose, untreated group. Changes were not observed in the remaining animals in the group, nor any other challenged group.

In the lung, at day 1 pi, changes were generally minimal (Fig. 5B), comprising slight bronchiolar, luminal, inflammatory cell exudation. On day 2 pi, similar exudates were observed in the high dose, treated and untreated, and the low dose, treated groups, with minimal, necrotising bronchiolitis in one animal in the high dose, untreated group. By day 2 pi, similar exudates were observed in the high dose, treated and untreated groups, and the low dose, treated group. In addition, minimal, necrotising bronchiolitis was seen in one animal in the high dose, untreated group. At days 4 and 5 pi, in the untreated groups, changes comprised bronchiolar luminal exudation, parenchymal mononuclear cell infiltration (Fig. 6C), bronchial gland necrosis (Fig. 6C, inset), and peribronchial and perivascular oedema (Fig. 6D). In the untreated groups, these changes were more severe (Fig. 5B). In oseltamivir-treated groups, changes were minimal to mild (Figs. 5B, and 6E, F), and bronchial epithelial and glandular necrosis were not observed. In both treated and untreated groups, there was a slight, dose effect in the frequency of pathological observations.

In the control animals, changes in the nasal cavity, trachea and lung were not observed.

## Discussion

This study determined the effect of reducing intra-nasal dose of infectious virus on the kinetics of virus shedding and disease progression, and compared the effects of oseltamivir treatment on ferrets infected with a high or low virus dose. Although Cal/04 induces a relatively mild disease in ferrets [9], [11], [25], [33] we were able to reliably infect with 100 pfu via the intra-nasal route (over 50 ferrets infected with this dose in a number of different studies in this laboratory have all sero-converted, shed virus in nasal washes, and shown clinical signs of disease). The most reproducible early sign of infection (other than virus shedding) was the innate immune cell count in nasal washes which typically rose 100-fold following infection and is consistent with previous data [34]. During influenza virus infection the nasal cavity cell population comprises mostly neutrophils and monocytes/macrophages, and represents activation of the innate immune system [28]. We observed that a reduction of 10,000-fold in virus inoculum (from 10^6^ pfu to 10^2^ pfu) led to a delay of 1 day in reaching peak cell count, suggesting a slower progression of the innate immune response when using the 10^2^ pfu challenge dose. Taken as a whole, the spectrum of clinical signs (including temperature rise, transient weight loss, sneezing, nasal discharge and inactivity) was clearly observed in the present study following inoculation with 10^2^ pfu Cal/04, and was only modestly reduced compared to a 10^6^ pfu inoculation. The most noticeable effect of using the lower dose was the delayed kinetics of infection, rather than any major amelioration of disease. A previous study by Smith *et al* compared doses of 10^5^ and 10^2^ pfu Cal/04 in ferrets, however no obvious clinical signs were observed in that study [33]. In agreement with the present study Smith *et al* showed a delay in peak virus shedding in the lower dose group, but nasal washes were not taken every day and therefore the studies are not directly comparable [33]. A novel observation made in the present study is that lowering the challenge dose does not lead to lower virus shedding, but leads to increased shedding both in terms of total virus shed over the course of the infection, and peak titre of shed virus. We hypothesise that the slower innate immune response associated with the lower virus challenge, as described above, allows increased virus accumulation in the nasal cavity. The trend towards increased peak virus titre with lower challenge dose is also shown in Figure 1 of Smith *et al*, although the authors did not highlight the observation [33].

We previously reported a low intra-nasal dose to demonstrate the efficacy of defective interfering influenza virus 244 in the ferret model [26], [27]. Here, oseltamivir was used as a test treatment with both high and low doses of Cal/04, as oseltamivir was used extensively in humans during the 2009 pandemic, and previous studies have demonstrated oseltamivir efficacy in ferrets against a number of influenza virus strains [12], [19], [20], [26]. Using nasal wash cell count and virus titre as measures of infection, it was noted that oseltamivir treatment had little effect on either measure following infection with 10^6^ pfu. A previous study [12] which used a 10^6^ pfu Cal/04 challenge did show reductions in nasal wash cell counts (but not virus shedding), but used a substantially higher dose of oseltamivir than the human-equivalent doses used here. However, in the present study, nasal wash cell count showed a delayed rise due to oseltamivir treatment (commenced before or after virus inoculation) following infection with 10^2^ pfu Cal/04. Similarly, virus titres showed significant reductions in response to oseltamivir treatment following infection with 10^2^ pfu Cal/04. Furthermore, only the 10^2^ pfu-infected, oseltamivir-treated animals showed a reduction in lung viral RNA load at 5 dpi and a reduction in pathological changes in the nasal cavity at 4 dpi. In the experiment in which the therapeutic dose of oseltamivir was used (10 mg/kg/day), no nasal signs (sneezing, nasal discharge) were observed at all in the treated 10^2^ pfu group. An unexpected finding was an increase in severity of changes in the nasal cavity in the high dose, oseltamivir-treated group, compared with the untreated, high and low dose groups. Whether this represented some animal or challenge variation could not be determined. However, these differences had disappeared by day 4 when the most severe changes were observed in the untreated, high-dose group. The effect of oseltamivir treatment on the virus-induced pathological changes was more clearly observable in the low dose group, in particular a delay in changes in the nasal cavity from 2 to 5 dpi.

The rate of virus clearance did not benefit from oseltamivir treatment, as virus shedding in oseltamivir-treated ferrets is higher than in untreated ferrets at 6 dpi in both high and low dose challenge groups (Figure 2A). This lack of improvement of clearance kinetics has been observed before [26], and is thought to be due to viral rebound following cessation of treatment at 5 dpi.

In summary, while oseltamivir treatment was of limited efficacy in the high dose challenge (10^6^ pfu) model (with reduction of virus shedding at 1 dpi; reduction of trachea viral RNA load on 4–5 dpi; reduction of lung pathology at 4–5 dpi), it was highly effective with the low dose (10^2^ pfu) model.

Although oseltamivir treatment has previously been noted to reduce influenza virus load in ferret lung tissue [19], to our knowledge this is first study to show the dramatic ∼2000- to 5000-fold reduction in virus RNA load in the trachea at 4 dpi. One day later on day 5, RNA loads in the trachea were clearly above background in at least some treated animals (Fig. 4B), and viral RNA was also detectable in the lungs of most animals (Fig. 4C). These data suggest that oseltamivir is effective in slowing the passage of the Cal/04 virus down the respiratory tract, regardless of virus dose. It should be noted that sample sizes were too small to estimate statistical significance of these findings. A future study using larger sample sizes should be conducted to investigate further the effects of oseltamivir on viral RNA load in the ferret respiratory tract.

We conclude that the low dose (10^2^ pfu Cal/04 virus) ferret model leads to improved sensitivity in demonstrating the efficacy of oseltamivir, and thus may be valuable in the future for the study of influenza therapeutics and vaccines.
